# The effectiveness of an immersive virtual reality and telemedicine-based cognitive intervention on prospective memory in Parkinson’s disease patients with mild cognitive impairment and healthy aged individuals: design and preliminary baseline results of a placebo-controlled study

**DOI:** 10.3389/fpsyg.2023.1268337

**Published:** 2023-10-19

**Authors:** Maria Stefania De Simone, Alberto Costa, Gaetano Tieri, Sara Taglieri, Giorgia Cona, Eleonora Fiorenzato, Giovanni Augusto Carlesimo, Carlo Caltagirone, Silvia Zabberoni

**Affiliations:** ^1^Department of Psychology, Niccolò Cusano University, Rome, Italy; ^2^IRCCS Santa Lucia Foundation, Rome, Italy; ^3^Virtual Reality and Digital Neuroscience Lab, Department of Law and Digital Society, University of Rome Unitelma Sapienza, Rome, Italy; ^4^Department of General Psychology, University of Padua, Padua, Italy; ^5^Department of Systems Medicine, Tor Vergata University of Rome, Rome, Italy

**Keywords:** prospective memory, Parkinson’s disease, healthy aging, training, immersive virtual reality, telemedicine

## Abstract

**Introduction:**

Prospective memory (PM) impairments have been extensively documented in individuals with Parkinson’s disease associated with mild cognitive impairment (PD-MCI) and in those with healthy aging. Considering how PM failure decreases individuals’ quality of life and functional independence in the activities of daily living, training to enhance this ability could be a prior target of intervention.

**Objective:**

Here, we aimed to present the study protocol and preliminary results of a novel immersive virtual reality (IVR) and telemedicine-based (TM) cognitive intervention focused on executive abilities (i.e., planning, shifting, and updating) to improve PM functioning in PD-MCI patients and healthy elderly individuals.

**Methods:**

Outcome measures, collected before, immediately after and 2 months after the intervention, included: (1) pre-post training changes in objective cognitive functioning, evaluated with tests assessing executive functions and PM; (2) pre-post training changes in subjective perception of memory functioning, psychiatric symptoms, autonomy in daily living and quality of life, evaluated using the appropriate scales; (3) usability, feasibility and users’ compliance with the proposed IVR and telemedicine program. The efficacy of this intervention compared to an active control condition is currently being evaluated in a randomized, double-blind controlled trial, which will be conducted on 30 eligible PD-MCI patients and 30 older adults.

**Results:**

Preliminary results concerning between-group comparisons of demographic and neuropsychological screening data show a good balance among the intervention groups considered in this study. The results also suggest good levels of usability, feasibility and acceptability, thus supporting the notion that our intervention can be used to promote cognitive functioning, even in people with cognitive decline.

**Conclusion:**

Considering the relatively low costs and easy accessibility to this program, it could prove valuable in primary prevention initiatives and early cognitive rehabilitation for dementia risk reduction.

## 1. Introduction

In addition to the main symptoms of Parkinson’s Disease (PD), patients frequently have significant cognitive dysfunctions that can be present even in the early stages of the disease and represent a high-risk factor for the development of dementia over time (Williams-Gray et al., 2007; Kehagia et al., 2010; Aarsland et al., 2017). Therefore, the concept of mild cognitive impairment (MCI) has also been introduced in the PD continuum of cognitive dysfunction (PD-MCI) to identify the borderline stage between normal functioning and dementia (Litvan et al., 2012, 2018). It has been estimated that PD-MCI can be detected in approximately 15%–25% of newly diagnosed patients (Aarsland, 2016), a percentage that increases from 20% to 57% of individuals 3–5 years after diagnosis (Williams-Gray et al., 2007) and that is significantly associated with the subsequent development of PD dementia at an annual rate ranging from 9% to 15% (Pedersen et al., 2013). Cognitive deficits have been widely recognised as primarily responsible for the decrease in patients’ quality of life and functional independence in the activities of daily living (Biundo et al., 2017a; Hering et al., 2018). Since therapeutic strategies aimed at counteracting cognitive decline in PD have thus far demonstrated limited efficacy, targeted non-pharmacological early interventions to improve cognitive functioning and even potentially prevent or delay the emergence of dementia are urgently needed (Biundo et al., 2017b).

Prospective memory (PM) deficits have been consistently indicated among the salient cognitive domains typically impaired in PD-MCI (i.e., attention/executive functions, memory, speed of processing and visuospatial ability) (Costa et al., 2014, 2018). PM is defined as the ability to form, maintain in memory and carry out delayed intentions at a certain time (time-based prospective memory) or when a specific event occurs (event-based prospective memory) (Costa et al., 2012, 2014). Therefore, PM is crucial for success in everyday life because of its large impact on multiple aspects of daily living, such as health (e.g., remembering to take medication at the right time or remembering to buy drugs when passing the pharmacy on the way home), social skills (e.g., remembering appointments at the proper time) and financial management (e.g., remembering to pay bills on time) (Jáger and Kliegel, 2008).

An increasing body of research findings support the notion that PM impairments in PD could be primarily related to deficits in executive functioning. For example, several studies demonstrated that PD patients tend to perform worse on PM tasks in which executive functions are particularly stressed, such as time-based versus event-based tasks or non-focal versus focal tasks (Katai et al., 2003; Costa et al., 2008; Foster et al., 2009). With regard to executive functions, it has been shown that a significant interaction among set-shifting, planning and updating deficits could represent a major factor in explaining the pattern of PM impairments in these patients (Kliegel et al., 2011; Costa et al., 2018; Maggi et al., 2019). This finding on the role of executive functions in PM has also been supported by research on cognitive rehabilitation, which has demonstrated significant improvement of PM in PD patients who undergo shifting abilities training (Costa et al., 2014) and external strategy potentiation for intention encoding (Foster et al., 2017). Considering the widely demonstrated fundamental role of the frontal lobes in executive functioning, this evidence collected at the behavioural level is consistent with PD neuropathology, which affects fronto-striatal circuitry extensively and early as a consequence of the depletion of dopamine in the basal ganglia (Kliegel et al., 2002).

PM failures have also been extensively documented in healthy aging as one of the most commonly reported memory difficulties. Similarly to PD, the deficit in this population has been linked to subtle age-related deterioration of executive processes, i.e., planning, inhibition, updating and shifting, which has been associated with the underlying frontal changes that occur during physiological aging (Cona et al., 2012). In line with this evidence, neuropsychological findings also suggest that PM in healthy aging could be improved by training focused on executive functions related to intention initiation and action execution phases of PM processes (Hering et al., 2014).

Considering the relevant impact of PM impairments on individuals’ autonomy in daily living activities and, thus, on their quality of life, training to enhance the ability to remember and execute delayed intentions could be an important priority for obtaining ecologically relevant improvements in populations that typically complain of these memory deficits, such as normal elderly subjects and PD-MCI patients. In this framework, we developed and are currently testing the efficacy of a novel cognitive training focused on executive abilities to improve prospective memory functioning in PD-MCI patients and healthy elderly individuals. Importantly, we chose to implement this cognitive training with a combined immersive virtual reality (IVR) and telemedicine (TM) approach. Compared to existing interventions in the literature, these technological improvements applied to cognitive training (i.e., IVR and TM) are new and innovative, whose expected advantages are mainly twofold. First, with respect to classical laboratory-settled cognitive training, the application of IVR has proved to be a promising methodology for improving the ecological validity of training. In fact, it offers a more ecological environment for training activities that can simulate events which patients might encounter in their daily lives; this could, in turn, enhance transfer effects of cognitive improvement to outcomes related to patients’ daily living. Moreover, it has also been shown that IVR induces the sense of presence (i.e., the illusion of being in the virtual space which allows eliciting realistic reactions to virtual stimuli) and thereby increases engagement, motivation and the feeling of entertainment in users (Sanchez-Vives and Slater, 2005; Tieri et al., 2018); this, in turn, could reduce attrition rates in training programs. Second, when TM is adopted training can be administered and performed remotely; in this sense, it represents an innovative approach for overcoming the obstacles associated with face-to-face intervention (e.g., related to the difficulty of keeping outpatient appointments due to work commitments, distance, lack of transportation or disability). Thus, this approach seems particularly suited for PD patients, considering that motor difficulties can potentially interfere with their participation in cognitive interventions, and this in turn could have the potential of reducing caregivers’ burdens.

We are currently evaluating the efficacy of this IVR and TM-based cognitive intervention, compared to an active control condition, in a longitudinal randomised controlled trial (RCT). Here, we present the rationale, study design and protocol of this RCT study, with the main aim of addressing the goal of transparency, quality, integrity, and reproducibility of research. Moreover, baseline clinical–neuropsychological features of the enrolled sample and preliminary data on usability are also presented.

## 2. Materials and methods

### 2.1. Study design

**Figure 1:**
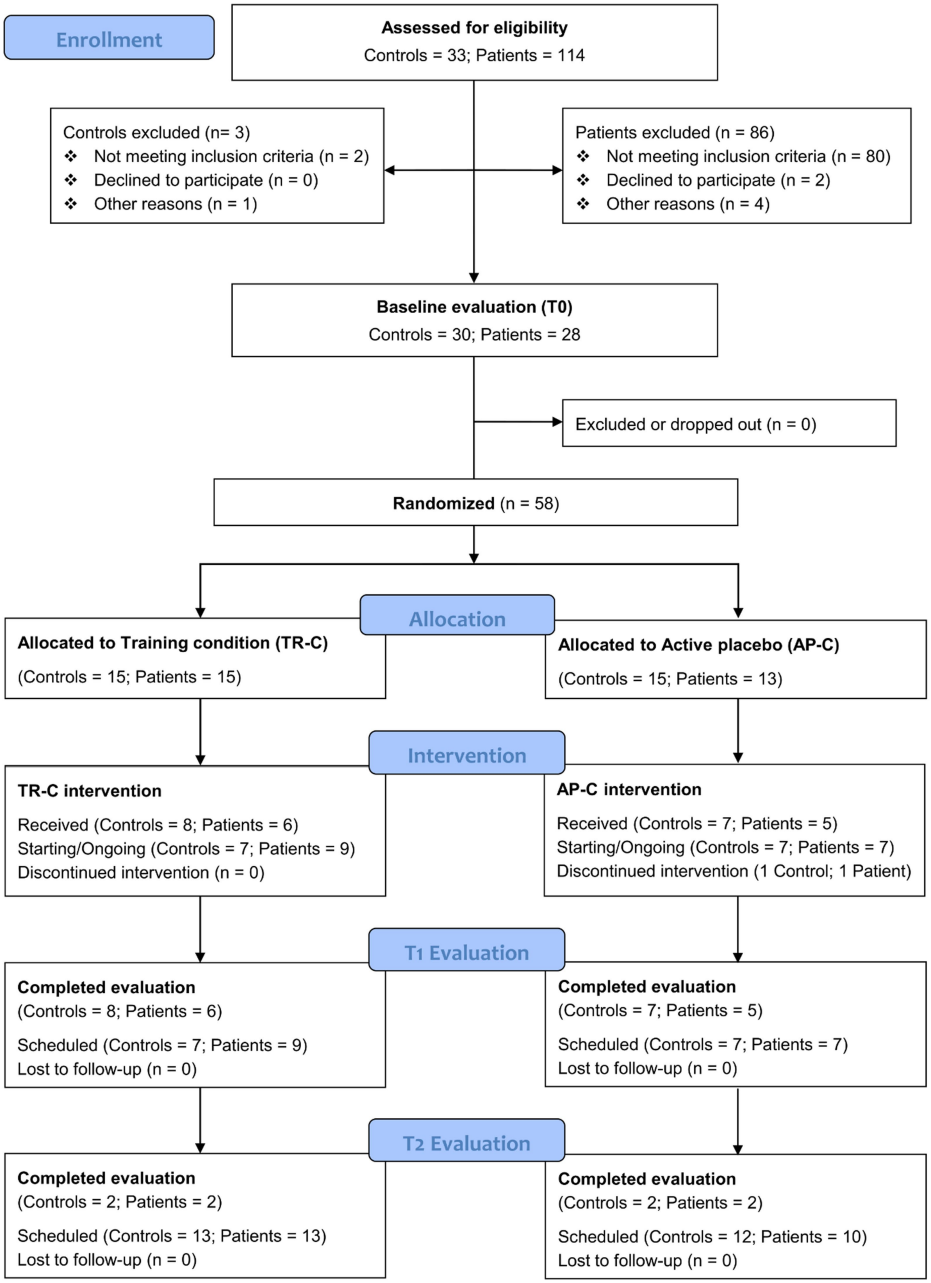
CONSORT flowchart.

This study is a double-blind RTC with a 4-parallel group design. It is being carried out in accordance with the Consolidated Standards of Reporting Trials (CONSORT) guidelines. The study design regarding enrolment and randomisation is presented in Figure 1. Briefly, after the eligibility criteria are checked in the screening phase and informed consent is obtained with a signature, the enrolled PD-MCI and HC subjects are invited to attend a baseline clinical and neuropsychological evaluation (T0) no more than 3 weeks prior to the intervention. Subsequently, to assess the superiority of the training compared with an active control, both PD-MCI and HC participants are randomly allocated to one of the two experimental conditions: Training condition (TR-C: 15 PD-MCI and 15 HC) and Active Placebo condition (AP-C: 15 PD-MCI and 15 HC), resulting in 4 parallel groups. For all four groups, 12 intervention sessions are carried out over a 4-week period (3 sessions/week, each lasting 30 min). Participants perform their respective intervention (TR-C or AP-C) at home using a dedicated virtual reality head-mounted display (HMD). By means of a telemedicine platform, developed *ad hoc* for the present intervention, we were able to remotely (1) assign the intervention for each participant and (2) control and monitor the intervention in real time, as well as transfer data using cloud-computing technology.

Before beginning the intervention, the participants are trained in the use of the HMD device during an in-person session. At the end of the 4-week intervention period, two clinical and neuropsychological follow-up assessments (T1: no more than 1 week after the intervention; T2: 2 months after the intervention) are scheduled.

This study is being carried out at two different locations in Italy, i.e., the IRCSS Santa Lucia Foundation of Rome and the Department of General Psychology and Padova Neuroscience Centre of the University of Padua. Each location is actively involved in participant recruitment, clinical/neuropsychological evaluations (T0, T1, and T2) and administration and monitoring.

The study is being performed in accordance with the ethical standards of the 2013 Declaration of Helsinki. The study was approved by the Ethic Committee of IRCCS Santa Lucia Foundation as well as by the Ethical Committee of the School of Psychology University of Padua. Written informed consent is collected for all participants during the screening evaluation. The principles of good clinical practice are being observed in conducting the study.

### 2.2. Objectives

The main objectives of the present work are threefold: (1) to assess whether the IVR and TM-based cognitive training, when compared to the active control condition, leads to post-intervention gains in objective cognitive functioning in general, and particularly in PM performance, in both PD-MCI and healthy elderly subjects; (2) to assess the transfer effect of the intervention on everyday-life abilities, mood and quality of life; (3) to investigate the users’ compliance with a telemedicine approach combined with IVR.

### 2.3. Participants

Recruitment for this study is currently ongoing. Both PD-MCI patients and HC subjects are equally recruited (target of each unit: 15 PD-MCI and 15 HC).

For the patients’ group, inclusion criteria are: (1) idiopathic PD, defined in accordance with the United Kingdom Parkinson’s Disease Society brain bank criteria (Postuma et al., 2015); (2) the presence of MCI (Litvan et al., 2012). In particular, for this study we include PD patients who perform at 1.5 standard deviations below the normative population on two tests of a comprehensive neuropsychological screening battery, one of which investigates executive functioning. Details about the tests used for diagnosis in the screening phase are reported in the subsequent section. Exclusion criteria are: (1) major psychiatric disorders or neurological conditions other than PD (e.g., depression, psychosis, behavioural disorders); (2) severe non-neurological comorbidities (e.g., cardiovascular, metabolic and/or endocrinological diseases); (3) severe sensory or motor disturbances liable to interfere with the intervention; (4) deep brain stimulation.

Patients have to be on stable treatment with levodopa and/or dopamine agonists before enrolment and during the entire observation period. Neuropsychological screening and outcome evaluations, as well as intervention sessions, are exclusively administered in the “on-therapy” condition (i.e., within 1 h after taking levodopa and/or dopamine agonists). During the 2-month follow-up, participants do not undergo any type of cognitive intervention.

For the control group, inclusion criteria are: (1) absence of neurological or psychiatric disorders; (2) no history of alcohol or drug abuse; (3) age and education-appropriate cognitive functioning as confirmed by performance above the normality cut-off scores on each neuropsychological test administered in the screening phase and a Mini-Mental State Examination score ≥ 26.

For both groups, additional inclusion criteria are the ability to give their written consent to participate in the study, normal or corrected to normal vision and hearing, native or fluent proficiency in Italian, ability to commit to the whole intervention and follow-up assessments.

### 2.4. Neuropsychological evaluation

#### 2.4.1. Screening battery

The tests comprising the extensive neuropsychological battery that is administered in the screening phase to assess the eligibility of both PD patients and healthy controls are described below according to the cognitive domains they examine: (a) Global cognition: Mini-Mental State Examination (MMSE, Measso et al., 1993) and Mini-Mental Parkinson State Examination (MMPSE, Costa et al., 2013); (b) Verbal episodic long-term memory: 15-Word List test (immediate and 15-min delayed recall) and Short story test (immediate and 20-min delayed recall) (Carlesimo et al., 1996, 2002); (c) Short-term memory and working memory: Digit span and Corsi Block Tapping test forward and backward (Monaco et al., 2013); (d) Attention and Executive functions: Phonological and Semantic word fluency test (Costa et al., 2014), Modified Card Sorting Test (MCST, Nocentini et al., 2002), Trail Making test parts A and B (TMT, Giovagnoli et al., 1996), Stroop Test (Barbarotto et al., 1998); (e) Reasoning: Raven’s Coloured Progressive Matrices (Carlesimo et al., 1996); (f) Constructional praxis: Copy of simple drawings and Copy of Drawing with landmarks (Carlesimo et al., 1996), Clock Drawing test (CDT, Caffarra et al., 2011); (g) Language: Naming subtest of the ENPA (NeuroPsychological Examination for Aphasia, Capasso and Miceli, 2001).

For all tests we use Italian normative data for score adjustment (sex, age and education) and to define normality cut-off scores, which were established as the lower limit of the 95% tolerance interval for a confidence level of 95%. For each test, normative data are reported in the corresponding references.

#### 2.4.2. Outcome measures

All participants are evaluated for cognition, subjective perception of memory functioning, psychiatric symptoms, autonomy and quality of life before (T0), immediately after (T1) and 2 months after the 4-week intervention period (T2).

First, to evaluate the effect of the training on cognition (PM and attention/executive functions), we compare the mean scores reported by each group at T0, T1, and T2 on the following outcome measures: (a) Prospective memory: Memory for Intention Screening Test (MIST, Raskin, 2009); (b) Planning abilities: Zoo map test (Wilson et al., 1998) and Tower of London test (Krikorian et al., 1994); (c) Shifting abilities: Trail Making Test (Giovagnoli et al., 1996) and Alternate Fluency task (Costa et al., 2014); (d) Updating abilities: updating subtest included in the Test of Everyday Attention (TEA, Zimmermann and Fimm, 1994). When possible, parallel forms (i.e., alternative versions using similar material) are adopted for the T1 and T2 follow-up visits in order to avoid learning and practice effects.

Additionally, training-induced changes in subjective perception of memory functioning, psychiatric symptoms, autonomy in daily life and quality of life are evaluated by comparing scores at T0, T1, and T2 reported in the following questionnaires/scales.

(a) Self-perception of memory functioning: Prospective and Retrospective Memory Test (PRMQ, Smith et al., 2000); (b) Psychiatric symptoms that typically appear in the early dementia stage (i.e., depression, anxiety and apathy): Beck Depression Inventory (BDI; Beck et al., 1961), State–Trait Anxiety Inventory (STAI, Spielberger, 1983) and Apathy Evaluation Scale (AES, Marin et al., 1991); (c) Autonomy: basic and instrumental activities of daily living scale (ADL, IADL, Lawton and Brody, 1969) as well as the Parkinson’s Disease – Cognitive Functional Rating Scale (PD-CFRS; Kulisevsky et al., 2012); (d) Quality of Life: Older People’s Quality of Life Questionnaire (OPQOL-35, Bowling, 2010), which is administered to HC subjects, and Parkinson’s disease Questionnaire (PDQ-39, Hagell and Nygren, 2007), which is administered to PD-MCI patients; (f) usability and acceptance of the proposed IVR and telemedicine-based program: a modified version of the System Usability Scale (SUS, Brooke, 1996; Grier et al., 2013).

### 2.5. IVR and TM apparatus

The virtual stimuli, objects and environment were designed by using 3DS Max 2018 (Autodesk, Inc.) and Unity 2019 game engine software,^1^ respectively.

The virtual environment consists of a life-size supermarket (22 m × 40 m), which consists of different aisles and shelves filled with some goods and tools. All 3d models of the goods and tools in the supermarket were designed to resemble real brands and packages from common products that can be found in Italian supermarkets (we did not include the name of the brand on the products in order to avoid any personal preferences). The virtual environment of the supermarket was used for all tasks included in the intervention (Planning, Updating, and Shifting – see description below).

The tasks were programmed by means of Unity 2019 and built for Oculus Go head mounted display (HMD), with a resolution of 1.440 by 1.280 (per eye), a refresh rate of 72 Hz and 3 degrees of freedom (DoF).

The interaction modality was thought up and programmed in order to offer the participants the easiest and most intuitive way of interacting with the virtual environment. It consisted of using the Oculus Controller, which combined with a customised C# script developed in Unity allowed pointing a ray-cast towards the virtual objects and interacting with them by pushing the trigger button. In this way, the participants could easily use their hand to drive the ray-cast and their index finger to push the button and make decisions.

Similarly, the Oculus Controller was also programmed for walking and navigating inside the virtual environment (as requested in the Planning task). In this case, participants were required to point the ray-cast towards the blue or green circles located on the supermarket floor and to click the trigger button to change their body position and orientation toward the circles; we designed and implemented this kind of “walking mode” because it is very intuitive and easy to follow and requires little space and small body movements. We avoided the use of more naturalistic methods, such as real walking, which would have required a large space around the participants. Using our design, the users are able to carry out the task while they are still and seated on a chair and without any caregiver supervision.

For remotely controlling and managing the HMD devices and sequences of the tasks in real time, we created a dedicated telemedicine platform; it consists in a web app, which was developed in C# (for the back-end framework) and in JavaScript (for the front-end framework).

### 2.6. Intervention

Both intervention conditions (i.e., Training and Active Placebo) were implemented in the IVR environment and administered in HMD using a telemedicine approach.

#### 2.6.1. Training condition (TR-C)

This consists of a cognitive IVR training focused on executive functions to improve PM. It is administered during a consecutive 4-week period (i.e., 3 sessions/week, each lasting 30 min), for a total of 20 sessions. The selection of the most appropriate cognitive activities to be trained was made on the basis of empirical findings which indicate that in both patients with PD-MCI and in HC PM impairments may be primarily related to executive dysfunctions and that training executive abilities might significantly improve PM performance in both populations (Cona et al., 2012; Costa et al., 2014; Foster et al., 2017). During each of the 30-min sessions, participants are immersed in the real-like virtual supermarket and have to perform three different tasks (10 min each per session), which target the following executive functions.

##### 2.6.1.1. Planning

**Figure 2:**
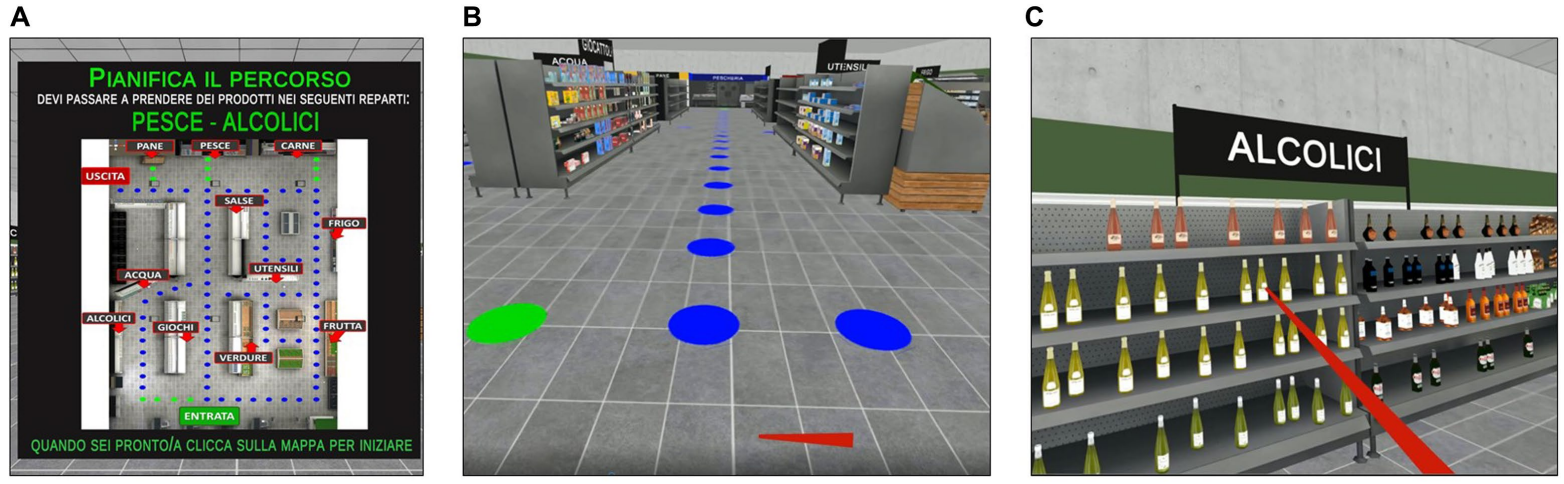
Planning task included in the training condition. *Planning Task*. **(A)** Map of the supermarket and task instructions (in Italian) displayed in the HMD in front of the participant. The map is used to plan a route for picking up a series of grocery items from the indicated locations in the supermarket aisles while following certain rules (which are displayed on the right side of the map). Translation of the instructions reported in **(A)**: “*Plan your* route *to pick up 2 products, one from the fish section and one from the alcohol section. When you are ready, please push the trigger button on the map.”* **(B)** Representation of the virtual supermarket. Blue and green circles on the floor indicate the steps of the path that participants can follow by pointing the ray-cast with the Oculus Controller and clicking the trigger button to move forward. **(C)** Example of picking the products by pointing the red ray-cast on the target and clicking the trigger button to pick it up.

The planning task was developed as an ecological, IVR-adapted version of the paper-pencil Zoo map test (Wilson et al., 1998). It is composed of two main scenes. Specifically, on the initial screen, participants are presented with the map of a supermarket (Figure 2A) and asked to plan a route that allows them to pick a series of target products from the indicated sections (e.g., “*You have to pick 2 products, one from the fruit section and one from the butcher section*”) while following certain rules. These rules include: starting at the entrance and ending at the exit, using the blue paths of the supermarket only once and the green paths as many times as needed. The task has been constructed in such a way that visiting the designated locations in the order in which they are given in the instructions can lead to errors; in this way, the initiative and the planning capacity of the participant are tested. In this phase, no time restriction is given for planning. After planning the route, and thus the order in which the indicated locations would be visited, the scenario changes and the participants are immersed inside the supermarket (Figure 2B). They can navigate through the aisles (which contain shelf units filled with grocery items) by using the HMD controller. Specifically, they have to place the ray-cast on the closest blue or green dot (depending on the path) on the floor and click the trigger button to move forward. At this point, the participants are asked to walk the route that they have previously planned. When approaching a designated location, they are asked to pick one product from the shelf. To pick an item, patients have to use the Oculus controller (i.e., by colliding the ray-cast with the product and clicking the trigger button to pick it) (Figure 2C). An acoustic signal indicates that the product has been correctly taken. At any time during the task the participants can see the map (in which their current position inside the supermarket is highlighted), and the rules and the products to pick by clicking on the Touch Pad button of their Oculus controller.

The planning task includes five levels of difficulty, each consisting of six different trials. Task difficulty increases gradually from Level 1 to Level 5 (by progressively varying the number of locations to visit and the number of blue and green paths that can be walked) depending on the participants’ performance. In fact, all participants start from the easiest Level 1. After completing three consecutive trials successfully (0 errors), they can increase their level of difficulty by one.

##### 2.6.1.2. Shifting

**Figure 3:**
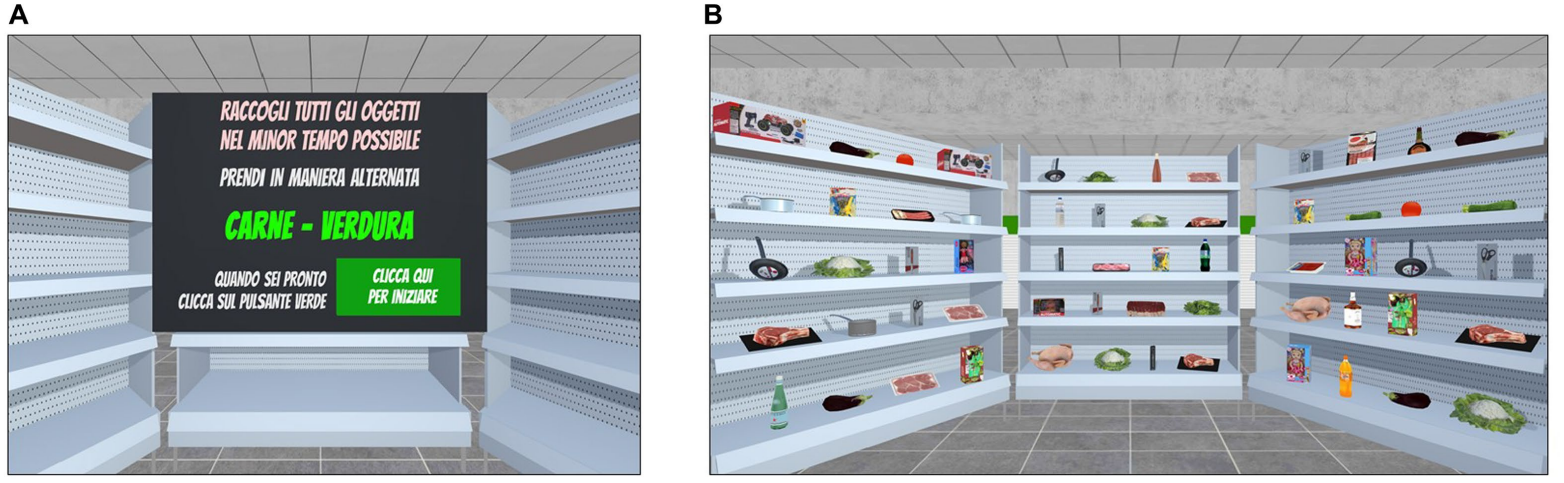
Shifting task included in the training condition. *Shifting*. **(A)** The Instruction panel (in Italian) displays the semantic categories of the target items to pick alternatively in the shortest time possible. Translation of instructions reported in **(A)**: “C*ollect the items in the shortest time possible. Alternately pick up meat items and vegetables items. When you are ready, please click on the green button.”* **(B)** Example of the participants’ point of view of the virtual shelves containing the products.

This task was developed as an ecological transposition in IVR of the traditional alternate fluency test (Costa et al., 2014). The scenario consists of three supermarket shelves filled with products that belong to two or more of the following semantic categories: fruits, vegetables, meat, fish, canned food, drinks, utensils, and toys (Figure 3B). At the beginning of the task, the participants are instructed to collect all the items from the shelves that belong to specific semantic categories (e.g., fruit and vegetables) in the shortest time possible while respecting the rule of continuously alternating the collection by shifting between the specified semantic categories. For example, the participants might be asked to collect all the items belonging to the fruit category and the vegetable category in a continuous alternating way; thus, to perform the task correctly, they should first pick a piece of fruit from the shelves and then a vegetable item, and thus continue to alternately pick items between the two semantic categories until all the required ones have been collected. To pick an item from the shelves, participants should place the ray-cast on it and click the trigger button for picking. An acoustic signal indicates to the participants that the product has been correctly taken. The semantic categories of the products to pick and the order in which they have to be collected are specified on the initial screen of each trial together with general instructions for carrying out the task (Figure 3A).

The shifting task includes 10 levels of difficulty, which vary in terms of (1) number of semantic categories to shift between, (2) number of products on the shelves and (3) the presence of products that do not belong to the semantic categories specified in the instructions (i.e., distractor items), which thus should not be collected if the task is to be completed correctly. Each level consists of six different trials. Participants start from the easier Level 1 and the exercises adaptively progress in difficulty according to their performance. In fact, once three consecutive trials are completed successfully (0 errors), the difficulty increases at the subsequent level.

##### 2.6.1.3. Updating

**Figure 4:**
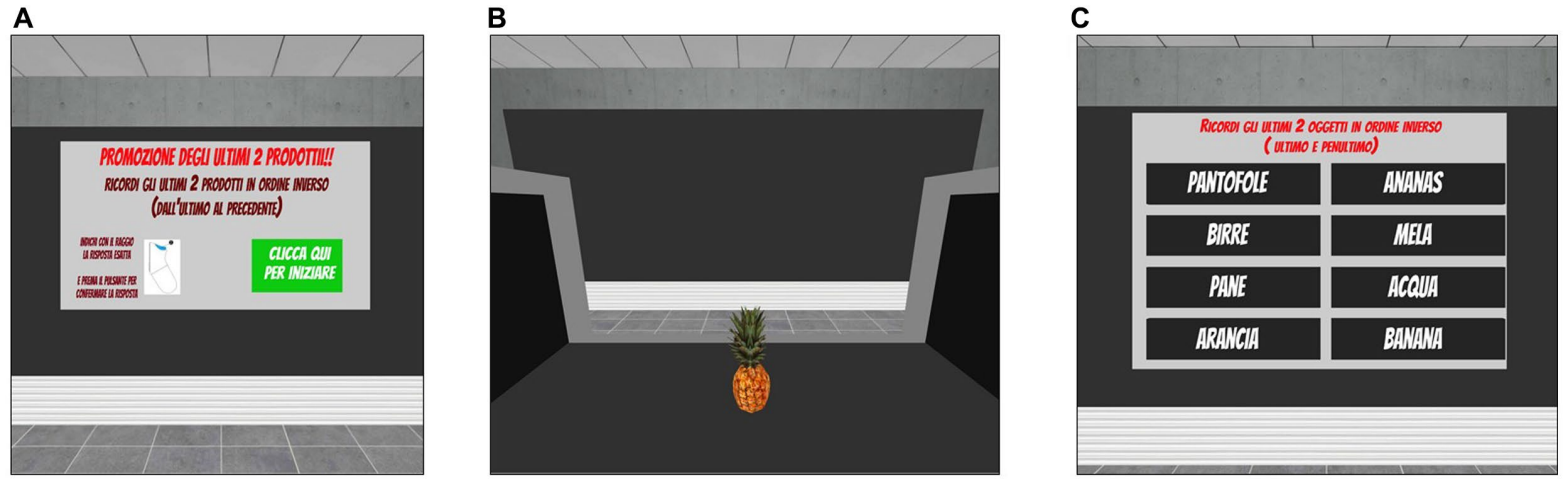
Updating task included in the training condition. *Updating*. **(A)** Instruction panel (in Italian) that indicates the number of products to remember (i.e., the last 2 products). Translation of instructions reported in **(A)**: “*Promotion of the last two products. Remember the last two products of the series in reverse order (i.e., last then next to last). When you are ready, please press the green button*.” **(B)** Example of the supermarket checkout, where participants could observe the products moving on the conveyor belt. **(C)** List of items from which the participants are required to identify the last *n* products of the previously presented series in reverse order by pointing the ray-cast towards the target item and clicking the trigger button to choose it.

This task was developed as an ecological and IVR-implemented version of traditional working memory tests. The scenario consists of a supermarket checkout, where participants are shown a series of goods and tools moving on a conveyor belt (one at a time, each shown for 4 s) (Figure 4B). The number of items included in each series randomly varies among trials and according to the level of difficulty. Participants are asked to name each item as it passes on the conveyor belt (i.e., to ensure that they are attending to the items) and to memorise the last *n* items of the series in reverse order (i.e., the last item, the next to last item, etc.). The number of items to be remembered in reverse order is specified on the initial screen of each trial, together with the general instructions for carrying out the task (Figure 4A). After completing the serial presentation of products, a virtual table appears in the scene in front of the participants that shows a list of possible items among which they have to identify the last *n* products of the previously presented series in reverse order. In this way, they are instructed to select from the alternatives the last product of the series as first, then the second to last item, and so on based on how many items they have to remember (Figure 4C). To make their selection, participants have to collide the ray-cast with their chosen alternatives and to click the trigger button to confirm. An acoustic signal indicates that the selection has been correctly acquired by the system. Since the number of items in each series is not fixed, but varies randomly among trials and levels of difficulty, here the ability to continuously update, maintain and manipulate information in working memory is particularly required to perform the task correctly.

The updating task includes 10 levels of difficulty, each consisting of six different trials. Task difficulty adaptively increases from Level 1 to Level 10 (by progressively varying the average length of the series and the number of items to be remembered in reverse order) according to the participants’ performance. In fact, all participants start from the easiest Level 1 and after completing three consecutive trials successfully (0 errors), they can increase their level of difficulty by one.

#### 2.6.2. Active placebo condition (AP-C)

The active placebo consists of three tasks (i.e., placebo-planning, placebo-shifting, and placebo-updating), which were developed using the same environments and settings described for the TR-C tasks. However, in this condition participants are required to virtually carry out daily actions by following prearranged instructions that make very low cognitive demands. In particular, in the *placebo-planning task* participants simply have to follow the instructions provided on the initial screen in order to produce a route in the supermarket without errors (the rationale behind this task is close to that of the Zoo map test version B, Wilson et al., 1998). In the *placebo-shifting task*, participants only have to pick all the items on the shelves as fast as possible. In the *placebo-updating task*, participants only have to remember the last item of each series of products that flow on the conveyor belt. Similarly to what has been previously described for the TR-C, these three placebo tasks are structured into several levels (i.e., the placebo-planning task includes five levels of difficulty, the placebo-shifting task and the placebo-updating task include 10 levels of difficulty), each consisting of six different trials. The difficulty of the task increases in an adaptive and progressive way from the first to the last level based on the participants’ successful completion of the three consecutive trials (0 errors).

Frequency and duration are the same as the TR-C (i.e., 3 sessions/week, each lasting 30 min, for a total of 20 sessions over a period of 4 weeks).

In both conditions (TR-C and AP-C), the administration order of the three tasks is randomised in each session. Progress and behavioural performance are monitored and recorded in real time through the telemedicine platform.

Prior to beginning the at-home intervention, all participants are trained in the use of the IVR HMD tool in an in-person session to account for any differences in baseline technological skills. During this session, participants see a series of tutorial videos and go through each exercise (according to allocation) until they feel familiar with the use of the device. Moreover, the participants are given an *ad hoc* user’s manual in paper format that provides detailed information about the HMD operating mode and activities to be carried out, as well as the contacts of the support team. At the end of the session, a dedicated IVR HMD is delivered to each participant so that they can perform the 4-week intervention at home. During the intervention at home, remote technical support is available if requested. Interventions are scheduled daily and monitored through the telemedicine platform.

### 2.7. Data analysis

#### 2.7.1. Planned statistical analysis

Statistical analyses that will be applied to the whole dataset of outcome measures include descriptive statistics (e.g., means, standard deviations, frequencies) and graphical exploratory data analytic techniques (e.g., residual plots, normal Q-Q plots) that will be performed to describe distributions of the data, to identify potential outliers and missing values, and to check for the possible violation of assumptions necessary for the planned statistical methods. Between-group differences in demographic (i.e., age, education) and baseline neuropsychological test performance will be assessed using parametric (independent t-test, one-way analysis of variance – ANOVA) or non-parametric tests (Mann–Whitney, Kruskal-Wallis) when appropriate. Chi-square test will be used to assess differences in sex distribution among groups. If data will be of normal distribution, the effect of the IVR and TM-based attention/executive training (Training vs. Active placebo) on outcome measures (T0 vs. T1 vs. T2) will be analysed using either repeated measures ANOVA or a similar repeated measures statistical technique such as linear mixed models. If linear model assumptions will be severely violated, an analogous non-linear repeated measures statistical approach will be used (e.g., non-parametric analysis or generalized mixed model underlying a count-like distribution). In this case, variable transformation may be performed if appropriate.

#### 2.7.2. Current statistical analysis

In this preliminary study, statistical analyses were performed using the Statistical Package for the Social Sciences (IBM SPSS, Version 22.0, Inc., Chicago, Illinois). To detect between group differences in demographic data (age, years of education, sex distribution), one-way analyses of variance (ANOVA) were run. Differences in gender distribution were assessed using a Chi-square test. To compare the performance of the two groups of PD-MCI patients (PD-MCI allocated to the TR-C and PD-MCI allocated to AP-C) on the tests included in the neuropsychological screening battery, independent sample t-tests with bias-corrected-accelerated (BCa) bootstrapping (1,000 resamples; Efron and Tibshirani, 1986; Wilcox and Rousselet, 2018) were performed to account for deviations from normality. Equal or unequal variances were considered for the t-tests based on Levene’s test results. The same statistical treatment was used to compare the neuropsychological screening data of the two groups of HC (i.e., HC allocated to the TR-C and HC allocated to AP-C). To account for multiple comparisons, the Benjamini–Hochberg procedure (Benjamini and Hochberg, 1995) was used to control the false discovery rate (FDR). Finally, as a preliminary examination of usability, a qualitative evaluation of frequency responses reported on the SUS scale by a sub-group of participants who had completed the intervention is reported.

## 3. Results

### 3.1. Demographic and neuropsychological screening data

As reported in Figure 1, to date a total of *n* = 114 patients with idiopathic PD and *n* = 33 potential controls have been screened for eligibility. After this stage, *n* = 86 patients and *n* = 3 controls were excluded because they did not meet the inclusion criteria or declined to participate, while the remaining *n* = 58 participants (*n* = 28 PD-MCI patients and *n* = 30 HC subjects) resulted eligible and were thus submitted to the baseline outcome evaluation (T0). Then, they were randomly assigned to one of the experimental conditions (i.e., TR-C or AP-C). Specifically, 15 PD-MCI and 15 HC were assigned to the TR-C, while 13 PD-MCI and 15 HC were assigned to the AP-C.

In the TR-C group, 14 participants have received and completed both the allocated intervention and the T1 evaluation (6 PD-MCI and 8 HC), 4 participants have also completed the T2 follow-up evaluation (2 PD-MCI and 2 HC), and 16 participants are going to start or are currently performing the 4-week intervention (9 PD-MCI and 7 HC).

In the AP-C group, 2 participants were excluded during the intervention (1 PD-MCI because he/she failed to follow the scheduled sessions and 1 HC because he/she reported feeling sick when using the HDM), 12 participants have received and completed both the allocated intervention and the T1 evaluation (5 PD-MCI and 7 HC), 4 participants have also completed the T2 follow-up evaluation (2 PD-MCI and 2 HC), and 14 participants are going to start or are currently performing the 4-week intervention (7 PD-MCI and 7 HC). We are currently involved in recruiting the remaining 3 PD-MCI patients (to be allocated to the AP-C) and 1 HC subject (to be allocated to the AP-C) and with the completion of the intervention administration and outcome evaluation at T1 and T2 for the entire experimental sample.

Demographic and neuropsychological screening data of the enrolled participants at baseline are reported in Table 1 according to the allocated intervention. As can be seen, the resulting four groups did not differ for mean age (F[3, 52] = 1.01, *p* = 0.39), years of education (F[3, 52] = 0.76, *p* = 0.52) or sex distribution (χ^2^[3] = 5.59, *p* = 0.13).

**Table 1:** Demographic and neuropsychological screening data of PD-MCI patients and HC subjects according to allocated intervention.

	Group			
	PD-MCI Training	PD-MCI Placebo	HC Training	HC Placebo
*Demographic*				
*n*	15	12	15	14
Age	70.5 (6.4)	65.6 (8.7)	69.7 (8.5)	68.9 (7.5)
Education	11.9 (4.0)	11.6 (3.3)	13.2 (4.1)	13.4 (3.8)
Sex	10 M: 5 F	9 M: 3 F	5 M: 10 F	8 M: 6 F
*General cognition*				
MMSE	28.5 (1.6)	27.2 (2.1)	29.1 (1.0)	29.3 (0.6)
MMPSE	29.0 (2.4)	26.8 (4.4)	29.4 (1.6)	30.1 (1.8)
*Memory*				
WL – immediate	36.7 (11)	32.1 (9.1)	47.2 (5.9)	42.6 (8.4)
WL – delayed	7.13 (3.1)	6.83 (2.9)	10.2 (2.1)	9.0 (1.80)
Prose – immediate	4.8 (1.9)	4.3 (1.6)	6.1 (1.2)	6.6 (0.9)
Prose – delayed	5.3 (1.3)	4.2 (2.1)	6.1 (1.0)	6.5 (1.5)
*Executive functions*				
TMT – A	51.9 (29.8)	68.6 (25.7)	40.1 (12.7)	39.7 (10.6)
TMT – B	151.6 (63.8)	264.5 (58.1)	101.2 (27.3)	114.0 (39.1)
MCST-criteria	4.1 (1.6)	3.1 (2.1)	5.7 (0.6)	5.9 (0.4)
MCST (p. errors)	4.2 (4.1)	7.0 (5.4)	0.8 (1.5)	0.3 (0.6)
Stroop test	18.5 (5.4)	12.9 (8.7)	23.9 (6.4)	24.1 (6.1)
Phonological fluency	35.0 (13.8)	25.6 (10.9)	39.1 (10.6)	40.6 (11.5)
Semantic fluency	44.1 (8.4)	34.4 (8.6)	50.1 (9.9)	51.5 (7.1)
*Short-term and working memory*				
Digit span forward	5.7 (0.8)	4.9 (0.9)	5.5 (1.1)	5.8 (0.8)
Corsi span forward	4.7 (0.8)	4.8 (0.9)	5.3 (1.0)	5.8 (1.2)
Digit span backward	4.5 (0.7)	3.3 (1.4)	4.8 (0.7)	4.5 (1.2)
Corsi span backward	4.4 (0.9)	3.7 (0.9)	4.6 (0.9)	5.1 (1.4)
*Reasoning*				
Raven’s matrices	28.3 (4.1)	28.3 (5.7)	32.1 (2.9)	33.1 (2.6)
*Constructional praxis*				
Simple drawings	9.1 (0.9)	8.6 (1.2)	10.2 (0.9)	10.4 (0.9)
Drawing + landmarks	63.8 (5.8)	62.3 (7.1)	66.6 (4.9)	64.9 (7.1)
Clock drawing	10.5 (2.4)	9.3 (2.2)	12.4 (0.7)	12.5 (0.5)
*Language*				
ENPA (nouns)	10	10	10	10
ENPA (verbs)	9.92 (0.3)	9.66 (0.5)	9.8 (0.4)	9.8 (0.4)

Mean scores with standard deviations in parentheses. PD-MCI, Parkinson’s disease patients with mild cognitive impairment; HC, healthy control; MMSE, Mini-Mental State Examination; MMPSE, Mini-Mental Parkinson State Examination; WL, word list test; TMT, Trail Making Test; MCST, Modified Card Sorting Test.

Regarding the neuropsychological screening data, no significant difference was detected in baseline neuropsychological scores between PD-MCI patients allocated to the TR-C and AP-C (FDR-adjusted *p* > 0.05 in all comparisons). Similarly, no significant difference in pre-intervention cognitive functioning was detected between HC participants allocated to the TR-C and AP-C (FDR-adjusted *p* > 0.05 in all comparisons).

### 3.2. Usability, feasibility and compliance of the IVR and TM-based intervention

The International Organization for Standardization (1998) defined usability as the “effectiveness, efficiency and satisfaction with which users achieve specified goals in a particular context of use” (Portney and Watkins, 2009). To evaluate these aspects, once participants have completed the at-home IVR and TM-based intervention they are administered a modified version of the System Usability Scale (Brooke, 1996). This is a brief, psychometrically valid and widely used global instrument for measuring user perceptions of a product’s usability (Bangor et al., 2008). This modified version includes 16 items, which are characterised by an alternating valence of positively-worded odd items and negatively-worded even items. Each item is scored on a 5-point Likert scale ranging from 1 (strongly disagree) to 5 (strongly agree). A global SUS score is calculated by using a published formula (Sauro and Lewis, 2011). Considering that to date only a subgroup of participants has completed the intervention (*n* = 15 HC and *n* = 11 PD-MCI), here we report a qualitative evaluation of the frequency responses to the main items included in the scale as preliminary data. Regarding the item “*I found the system simple to use*,” we found that 92% of the overall sample provided rates ranging from 4 to 5 on the Likert scale and only two participants provided a rate equal to 3 (8%). Considering the two groups separately, all PD-MCI patients (100%) and 13 HC subjects (86%) provided scores ranging from 4 to 5 on this item. Three items on the scale are aimed at investigating the possible appearance of adverse symptoms related to the use of the HDM tool (i.e., sickness, dizziness, fatigue). Specifically, regarding the item “*Did you experience discomfort linked to cybersickness?*,” we found that 96% of the overall sample disagreed or strongly disagreed with it (scores ranging from 1 to 2), and only one HC participant (4%) provided a score equal to 3. Regarding the item “*Did you experience dizziness?*,” we found that 88% of the overall sample disagreed or strongly disagreed with it (scores ranging from 1 to 2), whereas 3 participants (1 in the HC group and 2 in the PD-MCI group) were in agreement with the sentence (scores equal to 4). Regarding the item “*Did you struggle physically to wear the HDM tool for the entire duration of the training sessions?*,” we found that 92% of the overall sample disagreed or strongly disagreed with it (scores ranging from 1 to 2), and only 2 participants (1 in the HC group and 1 in the PD-MCI group) provided scores equal to 3.

Finally, regarding the attrition rate, which is considered a valid indicator of the acceptability of the intervention (Vita et al., 2022), we found that in the overall sub-sample of participants who have already completed the allocated intervention (*n* = 28, 16 HC and 12 PD-MCI), only 2 participants dropped out. Specifically, 1 HC subject allocated to the AP-C dropped out because of discomfort linked to the presence of cybersickness and 1 PD-MCI patient allocated to AP-C dropped out because he/she failed to follow the scheduled sessions.

## 4. Discussion

This RCT study is currently being carried out to evaluate the efficacy of a novel immersive virtual reality (IVR) and telemedicine (TM)-based cognitive intervention focused on specific attention/executive functions (i.e., planning, shifting and updating) to improve prospective memory (PM) abilities in both patients with PD-MCI and in healthy elderly controls.

The design of this study has many strengths with respect to previous interventions described in the literature. First, it includes an active control condition that mirrors the environment and structure of the training condition rather than a no-contact or wait-list control group comparison. This design has the advantage of allowing for greater control over the effects of intervening variables (e.g., expectations, additional stimulation from everyday routine); thus, any positive results can be more confidently attributed to the content of the program. Second, we developed a cognitive intervention that targets specific underlying cognitive processes, such as those that have been consistently associated with PM dysfunctions in both PD-MCI and HC (i.e., planning, updating and shifting) rather than more general cognitive functions (e.g., memory). From a technological point of view, another point of strength and novelty is the implementation of both intervention conditions (i.e., TR-C and AP-C) in IVR. Many multidisciplinary studies highlight that IVR is a valuable tool for the rehabilitation of cognitive and motor functions (Tieri et al., 2018). In fact, the technical properties of IVR have many advantages including high ecological validity, naturalistic interaction between user and virtual environment, sense of presence (i.e., the illusion of being physically present inside the virtual environment; Sanchez-Vives and Slater, 2005), active role of the user, 3D simulation of real-like events in safe situations, which could in turn have beneficial effects in boosting and maximising the potentially positive effects of the cognitive program. Furthermore, the importance of looking at the “generalisability” of the outcomes (i.e., transferring training-induced cognitive improvements from the laboratory to more complex situations in daily life) has been increasingly recognised and more efforts are necessary along these lines (Belleville et al., 2019). Thus, we designed virtual experiences that resemble those of everyday life in order to maximise the ecological validity and transfer effects of the cognitive tasks. Importantly, our IVR training includes an adaptive level of difficulty that can be modified according to the ability of each participant and, thus, enhance motivation and avoid frustration. A final point of strength is the use of a telemedicine approach. A telemedicine procedure that people can adopt directly in their own homes with remotely-controlled virtual reality HMDs will constitute an important step forward for distance treatment and will make a valuable contribution towards improving the continuity of care. In fact, telemedicine is an innovative way of overcoming the obstacles associated with face-to-face intervention in that it is a cost-effective, accessible, flexible and comprehensive choice for individuals who have difficulty keeping outpatient appointments due to work commitments, distance, lack of transportation and disability. Moreover, it can allow people who live in areas where there are few health infrastructures to join the intervention and commit to it for the duration; indeed, this would help achieve the sustainable goal of access to primary prevention and rehabilitation programs in underserved areas.

We expect that at the end of the treatment the participants in the overall training intervention condition (TR-C) will show better cognitive performances on tasks assessing executive functions and PM compared to those in the active placebo intervention condition (AP-C). In this context, we expect that improvements in outcome cognitive measures will be greater in the PD-MCI group than in the HC group since these patients typically present significant attentional/executive deficits, and thus also PM-related impairments, as a consequence of fronto-striatal circuits dysfunctions (Kliegel et al., 2008). Moreover, based on the evidence that IVR could be a feasible solution for enhancing the ecological validity of trained activities, we expect that cognitive training will have significant transfer effects on the functional abilities of daily living, subjective perception of cognitive functioning, mood and quality of life. Finally, we expect that both groups of participants will show a good level of compliance with the telemedicine-delivered intervention. This hypothesis is based on the reported good usability and feasibility of this approach (Tieri et al., 2018).

As preliminary results, the comparisons of demographic and neuropsychological screening data showed a good balance among the four interventional groups considered in this study. This finding supports the appropriateness of the randomisation procedure adopted in light of the relatively small sample included in the study. Moreover, although preliminary, the results of both groups of participants on the SUS scale overall suggest good levels of feasibility and usability, which supports the notion that our intervention could also be used to promote cognitive functioning in people with cognitive decline. Further evidence in support of the acceptability of the proposed cognitive program is the low number of drop-outs from the intervention up until now. In fact, as reported above, out of 16 HC participants who have already completed the intervention only one dropped out because of discomfort linked to cybersickness. Similarly, out of 12 PD-MCI patients who have already completed the intervention only one dropped out because he/she failed to participate in the scheduled sessions. Previous studies have documented that adverse effects (e.g., sickness, dizziness) might often be experienced by IVR users and that these symptoms are even more likely in elderly people and Parkinsonians (Kim et al., 2017); this, in turn, might result in reducing its use in clinic practice. However, our preliminary data seem to suggest that our IVR intervention, as it was originally designed and implemented (e.g., number of IVR sessions for week, task duration, scenario construction, safety “walking mode”) might work effectively in reducing the adverse effects often related to the IVR experience. Another main obstacle in the field of cognitive interventions is attrition rates, which are often high in training programmes (Willis and Belleville, 2016). This problem has been generally related to a lack of motivation to participate in the assigned training, which is often due to low pleasantness and attractiveness of the activities to be carried out. In this regard, the low attrition rate we recorded in our study could be associated with the use of IVR, which increases engagement, motivation and the feeling of entertainment in users as it induces a sense of presence (i.e., the illusion of being in the virtual space, which allows eliciting realistic reactions to virtual stimuli) (Sanchez-Vives and Slater, 2005; Tieri et al., 2018). Although more data are needed to build stronger evidence, this seems to be a good starting point for approaching the treatment of PD-MCI patients and healthy elderly individuals with IVR.

In summary, the final results of this RCT study will provide new evidence on the efficacy of an innovative cognitive intervention that integrates IVR and telemedicine in both PD-MCI patients and healthy aging individuals. Considering the relatively low costs and easy accessibility of this approach, it could make a valuable contribution to primary prevention initiatives and early cognitive rehabilitation to reduce the risk of dementia.

## Data availability statement

The raw data supporting the conclusions of this article will be made available by the authors, without undue reservation.

## Ethics statement

The studies involving humans were approved by the Ethic Committee of IRCCS Santa Lucia Foundation and by the Ethical Committee of the School of Psychology University of Padua. The studies were conducted in accordance with the local legislation and institutional requirements. The participants provided their written informed consent to participate in this study.

## Author contributions

MS: Conceptualization, Formal analysis, Investigation, Methodology, Writing – original draft. AC: Conceptualization, Supervision, Writing – review & editing. GT: Software, Visualization, Writing – review & editing, Methodology. ST: Data curation, Investigation, Writing – review & editing. GC: Data curation, Writing – review & editing, Supervision, Resources. EF: Data curation, Investigation, Resources, Writing – review & editing. GAC: Writing – review & editing. CC: Writing – review & editing. SZ: Conceptualization, Funding acquisition, Methodology, Supervision, Writing – review & editing, Resources.

## Footnotes

^1^ https://unity.com/
